# Combining long-lasting insecticidal nets and indoor residual spraying for malaria prevention in Ethiopia: study protocol for a cluster randomized controlled trial

**DOI:** 10.1186/s13063-016-1154-2

**Published:** 2016-01-12

**Authors:** Wakgari Deressa, Eskindir Loha, Meshesha Balkew, Alemayehu Hailu, Taye Gari, Oljira Kenea, Hans J. Overgaard, Teshome Gebremichael, Bjarne Robberstad, Bernt Lindtjørn

**Affiliations:** School of Public Health, Addis Ababa University, Addis Ababa, Ethiopia; School of Public and Environmental Health, Hawassa University, Hawassa, Ethiopia; Aklilu Lemma Institute of Pathobiology, Addis Ababa University, Addis Ababa, Ethiopia; Norwegian University of Life Sciences, Ås, Norway; Institut de Recherche pour le Développement (IRD), Maladies Infectieuses et Vecteurs, Ecologie, Génétique, Evolution et Contrôle (MIVEGEC), Montpellier, France; Department of Entomology, Faculty of Agriculture, Kasetsart University, Bangkok, Thailand; Center for International Health, University of Bergen, Bergen, Norway

**Keywords:** Cluster randomized controlled trial, Cost-effectiveness, Incidence, Indoor residual spraying, Long-lasting insecticidal nets, Malaria, Prevention and control, Ethiopia

## Abstract

**Background:**

Long-lasting insecticidal nets (LLINs) and indoor residual spraying (IRS) are the main malaria prevention interventions in Ethiopia. There is conflicting evidence that the combined application of both interventions is better than either LLINs or IRS used alone. This trial aims to investigate whether the combination of LLINs (PermaNet 2.0, Vestergaard Frandsen, Lausanne, Switzerland) with IRS using propoxur will enhance the protective benefits and cost-effectiveness of the interventions against malaria and its effect on mosquito behavior, as compared to each intervention alone.

**Methods/Design:**

This 2 x 2 factorial cluster randomized controlled trial is being carried out in the Adami Tullu district in south-central Ethiopia for about 116 weeks from September 2014 to December 2016. The trial is based on four arms: LLINs + IRS, LLINs alone, IRS alone and control. Villages (or clusters) will be the unit of randomization. The sample size includes 44 clusters per arm, with each cluster comprised of approximately 35 households (about 175 people). Prior to intervention, all households in the LLINs + IRS and LLINs alone arms will be provided with LLINs free of charge. Households in the LLINs + IRS and IRS alone arms will be sprayed with carbamate propoxur once a year just before the main malaria transmission season throughout the investigation. The primary outcome of this trial will be a malaria incidence based on the results of the rapid diagnostic tests in patients with a fever or history of fever attending health posts by passive case detection. Community-based surveys will be conducted each year to assess anemia among children 5–59 months old. In addition, community-based malaria prevalence surveys will be conducted each year on a representative sample of households during the main transmission season. The cost-effectiveness of the interventions and entomological studies will be simultaneously conducted. Analysis will be based on an intention-to-treat principle.

**Discussion:**

This trial aims to provide evidence on the combined use of LLINs and IRS for malaria prevention by answering the following research questions: Can the combined use of LLINs and IRS significantly reduce the incidence of malaria compared with the use of either LLINs or IRS alone? And is the reduced incidence justifiable compared to the added costs? Will the combined use of LLINs and IRS reduce vector density, infection, longevity and the entomological inoculation rate? These data are crucial in order to maximize the impact of vector control interventions on the morbidity and mortality of malaria.

**Trial registration:**

PACTR201411000882128 (8 September 2014).

## Background

Despite remarkable achievements in the fight against malaria over the last decade, there is still an unacceptably high level of malaria burden worldwide. In 2013, there were an estimated 198 million cases and 584,000 deaths, of which 80 % of the cases and 90 % of the deaths occurred in sub-Saharan Africa (SSA) [1]. The World Health Organization (WHO) recommends the universal coverage of the population at risk with long-lasting insecticidal nets (LLINs) [2] and targeted indoor residual spraying (IRS) with insecticide [3] for the control and ultimate elimination of malaria. In addition, IRS has been recommended to be scaled-up for malaria control across the different malaria endemicities including high transmission settings in SSA [4]. This has brought a significant shift from past practices in which IRS was limited for the prevention and control of malaria epidemics, particularly in unstable and seasonal malaria settings [5, 6].

Both LLINs and IRS have been shown to be effective in reducing malaria transmission when applied independently [7–9]. As a result, both interventions are widely applied for malaria prevention in many countries [10]. Of the 45 countries in Africa with ongoing malaria transmission, 38 adopted both the WHO’s policy of universal coverage with LLINs to populations at risk and IRS with insecticide [1]. In an effort to accelerate the control and ultimate elimination of malaria, IRS in combination with LLINs has also been deployed in the same geographical areas in 31 African countries [1]. The available evidence suggests that the joint intervention of the LLINs and IRS should be scaled up and that the combined effect of these interventions should be further evaluated [11–13].

Despite an increasing interest in the simultaneous use of both interventions, there are currently no clear guidelines on how these interventions should be combined [14]. At the same time, there is also a paucity of evidence as to whether their combined use is more effective in reducing the incidence of malaria than using either intervention alone [8, 15–17]. It is poorly understood how the interventions interact to improve malaria control. A few non-randomized observational studies and mathematical modelling exercises suggest a modest effectiveness, or conflicting results, when combining interventions for malaria reduction compared to either intervention alone [11, 12, 15, 17, 18].

Evidence on the effect of the combined use of LLINs and IRS from community-based field trials is conflicting. Consequently, it is difficult to draw any conclusions on whether the combination of IRS and LLINs is beneficial against malaria compared to one of the interventions alone. A recent review indicated that only one of the four published randomized controlled trials showed additional protection against fighting malaria when the use of LLINs was combined with IRS, compared to either method alone [14]. A multi-intervention trial in Benin showed no significant reduction in clinical malaria in children under 5 years of age from houses sprayed with bendiocarb in combination with LLINs, compared to children in houses with LLINs alone [19]. Similarly, in The Gambia a combination of IRS using dichloro-diphenyl-trichloroethane (DDT) and universal coverage of LLINs showed no added protection against malaria among children of 6 months to 14 years compared to the universal coverage of LLINs alone [20]. By contrast, a recent cluster randomized controlled trial in Tanzania, where the usage of LLINs was less than 50 %, reported some evidence of added protection against malaria infection in children 6 months to 14 years from the combining of insecticide-treated nets (ITNs) and IRS with bendiocarb compared to ITNs alone [13]. Lines and Kleinschmidt [21] recently discussed the design issues in conducting studies involving the combination of malaria vector control interventions and recommended further evidence from well-designed trials.

Good evidence on the effectiveness, costs and cost-effectiveness of malaria interventions will provide important information for decision-making in policy formulation, the revision of existing policy and/or the selection of optimal packages of interventions. Since the cost of both LLINs and IRS is greater than the cost of either intervention alone [22], it is important to estimate and evaluate whether the potential extra protection gained by combining both interventions represents good value compared with the added costs. This is particularly important in countries in SSA, where a scarcity of resources is the main impediment to malaria control. A recent review of the evidence of the costs and consequences of large-scale vector control for malaria concluded that both LLINs and IRS are highly cost-effective vector control strategies even though the former method has been identified as more cost-effective than the latter [22].

Malaria is a major public health problem in Ethiopia with approximately 65 % of its 90 million population living in areas at risk of malaria infection [23]. Mainly due to altitudinal and climatic features in most parts of the country, malaria transmission is seasonal and epidemic [24, 25]. The most important malarial parasites in the country are *Plasmodium falciparum* (60 %) and *P. vivax* (40 %) [23]. *Anopheles arabiensis* is considered the main malaria vector in the country, with *An. pharoensis* being a secondary vector.

The National Strategic Plan for malaria prevention and control in Ethiopia aims at scaling up and sustaining both LLINs and IRS interventions in malaria endemic areas [26, 27]. LLINs and IRS are applied either separately or in combination, and during the period from 2005–2011, more than 43 million LLINs were freely distributed to all households in malarious areas [28]. The Malaria Indicator Surveys (MIS), conducted in 2007 and 2011, revealed that 65 % and 55 % of surveyed households had at least one LLIN, respectively, whereas the reported use by children under 5 years of age, during the night prior to the survey, within households with at least one net ranged from 60 % in 2007 to 65 % in 2011 [29, 30].

In Ethiopia, the increased resistance of *P. falciparum* to chloroquine and sulfadoxine-pyrimethamine necessitated a change as the first-line antimalarial drug for the treatment of *P. falciparum* [31–33]. Consequently, artemether-lumefantrine (AL, Coartem®, Novartis, Basel, Switzerland) has been used as a first-line treatment for uncomplicated *P. falciparum* infection since 2004 [31]. Several studies have shown that AL remains highly efficacious against the treatment of uncomplicated falciparum malaria and with no report of adverse effects [34–37].

Several studies from Ethiopia have shown a high insecticide resistance in malaria mosquitoes, especially in relation to DDT, malathion, permethrin and deltamethrin [38–42]. DDT was the primary insecticide of choice for IRS in the country for a long time until it was replaced by deltamethrin in 2009. Unfortunately, resistance to deltamethrin was reported to be very high [38]. As a result, the National Malaria Control Program has adopted the use of bendiocarb and propoxur insecticides belonging to the carbamate family since 2012 [27, 43]. Currently, bendiocarb and propoxur are the primary insecticides of choice for IRS in Ethiopia. Bendiocarb and propoxur are carbamate insecticides evaluated and approved by the WHO Pesticide Evaluation Scheme, both of which have the potential to control pyrethroid-resistant mosquitoes [44].

This study aims to investigate the effect of combining LLINs and IRS with propoxur for the incidence of clinical malaria and the cost-effectiveness of the interventions against malaria and their effect on mosquito behavior, as compared to each intervention alone. The overall aim is to provide information for national and global policy-makers in their pursuit of improving malaria control by evaluating resource demands and the combined effect of both interventions on malaria. This protocol discusses the rationale for the choice of the interventions and describes the designs and methodological approaches being used to determine the effect of each intervention, in addition to evaluating the effect of the interventions.

A 2 x 2 factorial cluster randomized controlled trial was chosen to exploit the robustness of this design to help ascertain the efficacy of the combined interventions compared to either interventions alone and the standard routine practice. This protocol was developed according to the guidelines of the Consolidated Standards of Reporting Trials (CONSORT) statement extension for cluster randomized trials [45]. The scientific value of the inclusion of the control group in this trial was also extensively debated, as it was believed that this arm allows a more reliable comparison between the groups: LLINs + IRS versus LLINs alone or control, LLINs + IRS versus IRS alone and LLINs alone versus IRS alone or control. This is the most robust design in regard to cluster randomized trials to help ascertain the efficacy of the interventions. This study design is also believed to make the results more generalizable and applicable in resource-constrained countries.

### Trial objectives

#### Primary objective

The primary objective of this intervention study is to determine whether the combined use of LLINs and IRS with propoxur provides additional protection against malaria (*P. falciparum* and/or *P. vivax*) among all age groups in the study area compared to LLINs or IRS alone.

#### Secondary objectives

In the same study population, the secondary objectives of the trial are to:

1. Estimate the costs of LLINs + IRS, LLINs or IRS alone compared to the current routine practice, and to evaluate the incremental costs, effects and cost-effectiveness of interventions
2. Assess whether LLINs + IRS reduce entomological parameters, i.e., human biting rates, mosquito resting density, longevity, sporozoite rates, and the entomological inoculation rate (EIR) inside houses compared with LLINs or IRS alone
3. Determine whether LLINs + IRS improves the hemoglobin (Hb) concentration and reduces anemia among children under 5 years of age compared with children in LLINs or IRS alone

## Methods/Design

### Study setting

**Fig. 1:**
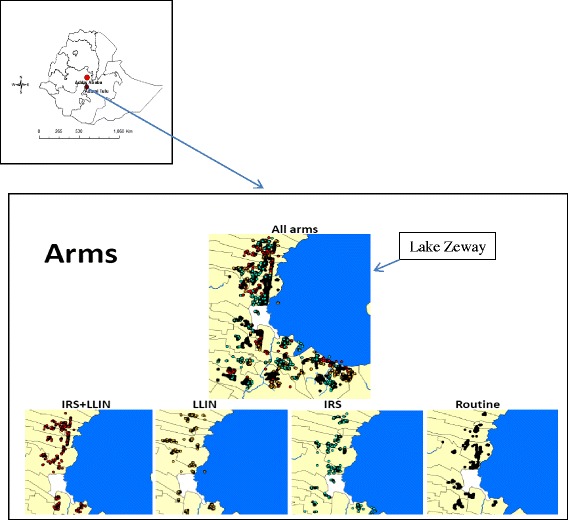
Map of the study area showing location of clusters in the study arms in Adami Tullu district

This study is being carried out in the Adami Tullu part of the Adami Tullu-Jiddo-Kombolcha *woreda* (hereafter referred to as the Adami Tullu district) in the East Shewa Zone of the Oromia Regional State in Ethiopia. The *woreda* (or district) is a local administrative unit in the country, followed by *kebeles* (the lowest government administrative unit, which is further divided into *gares*, or villages). The capital of the district, Zeway (or Batu), has a latitude and longitude of 7°56′N 38°42′E with an elevation of 1640 m above sea level. It is located approximately 160 km south of Addis Ababa along the highway connecting Addis Ababa to Nairobi via Hawassa. The district is set in the Great Rift Valley in south-central Ethiopia, with altitudes ranging from 1500 m to 2300 m. Administratively, the Adami Tullu district has 48 *kebeles*, each with an average population size of approximately 1000 to 5000 people. Figure 1 shows the geographical location of the study district and the description of the study arms in relation to Lake Zeway. The total annual rainfall is approximately 700 mm, with peaks during the main rainy season in July (250 mm) and August (220 mm). The mean minimum and maximum annual temperatures are 14.5 °C and 27.7 °C, respectively.

Based on the 2007 National Census [46], the projected population size of the district for 2014 was about 173,000 people and the population of Zeway town about 60,000. The main ethnic group is the Oromo, and the predominant religion is Islam. The majority of the population live in rural areas in houses made with mud or cement walls and thatched or iron roofs. Local residents primarily depend on farming, livestock rearing, and to a lesser extent on fishing in Lake Zeway for their subsistence. In 2014, there were one public and one non-governmental organization hospital, nine public health centers and 43 health posts in the district. The health centers are primarily staffed by health officers, nurses, midwives, pharmacists and laboratory technicians. Each *kebele* is intended to have at least one health post staffed by two health extension workers (HEWs) reporting to the health center.

Malaria is a leading health problem in the district. Transmission is seasonal and unstable, with several recorded epidemics of varying degrees [47, 48]. The main malaria transmission season occurs between September and December each year following the heavy rainfall between July and August, whereas the smaller peak occurs during May and June each year following small rains during March and April. Lake Zeway, which has many swampy areas, profoundly contributes to mosquito breeding in the study setting. *P. falciparum* and *P. vivax* co-exist in the area in varied proportions [47, 49]. A longitudinal community-based study carried out in 1994 revealed a malaria prevalence of 6.8 % (66 % *P. falciparum*, 31 % *P. vivax* and 3 % *P. malariae*) from July to December, peaking in September at 12.6 % [50]. Community-based cross-sectional surveys conducted in October to November 2006 and April 2007 indicated an overall parasite prevalence of 4.8 %, varying between localities from 1.7 % to 10.4 %, with 88 % *P. vivax* and 12 % *P. falciparum* parasite species composition [49]. Artemether-lumefantrine (AL, Coartem®, Novartis, Basel, Switzerland) and chloroquine are the first-line antimalarial drugs for the treatment of uncomplicated falciparum and vivax malaria, respectively. Both LLINs and IRS are the two major malaria preventive interventions implemented by the District Health Office (DHO).

*An. arabiensis* is the major malaria vector in the district and *An. pharoensis* is considered to have an auxiliary role [50]. An entomological study in the past showed that the former species prevailed from June to October with peak densities from July to September, while the latter species peak months were from September to November [50]. Preliminary insecticide susceptibility tests showed that *An. arabiensis* was greatly resistant to deltamethrin (mortality 14 %), alphacypermethrin (less than 1 % mortality), lambdacyhalothrin (4 % mortality) and permethrin (17 % mortality), but susceptible to bendiocarb and propoxur (mortality 100 %). *An. pharoensis* was fully susceptible to all the aforementioned insecticides with a 100 % mortality (*MalTrials* unpublished pilot data). Adami Tullu district has been one of the sentinel sites for the study of malaria epidemiology and entomology in Ethiopia due to its relatively higher malaria burden [33, 49–52].

### Design

This 2 x 2 factorial cluster randomized controlled trial, called *MalTrials*, will be carried out for approximately 116 weeks from September 2014 to December 2016. The village (or cluster) will be the unit of randomization, and an equal number of villages will be randomized to one of the four arms: (1) LLINs + IRS, (2) LLINs alone, (3) IRS alone or (4) control (routine practice). The control arm will receive the routine standard practice of malaria prevention of the Ethiopian Malaria Control Program.

### Participants

**Fig. 2:**
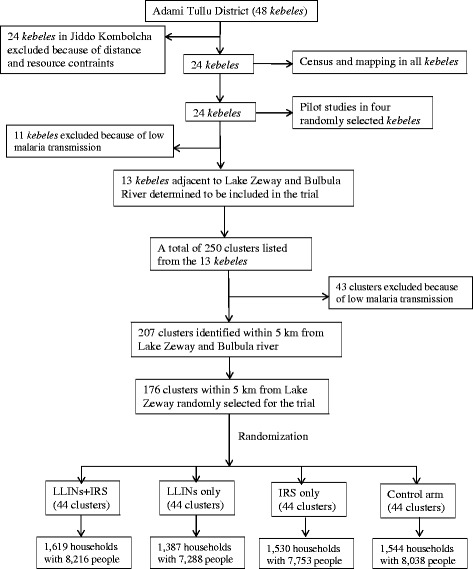
Flow chart of the study

This trial is only being conducted in the rural communities of the district. The reason for focusing on rural communities is due to the prioritization of IRS for malaria prevention in these areas. Prior to implementing intervention and randomizing villages to arms, a census, mapping, and pilot studies were carried out to estimate an optimum sample size (Fig. 2).

### Village inclusion criteria

Villages with a relatively easy access, relatively higher malaria transmission and located within 5 km from Lake Zeway will be included in the study. The preliminary findings indicated that the incidence of malaria was 8 cases per 10,000 person-weeks of observation for villages within 5 km from Lake Zeway, compared to villages beyond 5 km from the lake where the incidence rate was 0.5 cases per 10,000 person-weeks of observation (*MalTrials* unpublished pilot data).

### Village exclusion criteria

Villages with difficult access, a very low malaria transmission, and located beyond 5 km from Lake Zeway will be excluded.

### Participant inclusion criteria

All consenting residents of households in all clusters will be recruited for the study.

### Participant exclusion criteria

Residents and household heads who are not able to provide informed consent will be ineligible to take part in the trial.

### Randomization

From a total of 48 rural *kebeles* in the Adami Tullu district, 13 *kebeles* with a relatively higher malaria transmission adjacent to Lake Zeway were included in the study following a census carried out in 24 *kebeles* and pilot studies in four *kebeles*. From the total list of the clusters in 13 *kebeles*, 207 were located within 5 km of Lake Zeway, had a relatively higher malaria transmission, and were included in the sampling frame, of which 176 were randomly selected. The randomly selected clusters were numbered and equally randomized into the four arms following a computer-generated list using SPSS software, with a flow chart of the study given in Fig. 2. While the study is done in Ethiopia, randomization was done in Bergen in Norway. This was done to prevent selection bias by concealing the allocation sequence from the field researchers assigning villages to the four intervention groups until the moment of assignment. Thus, a researcher not involved in the study randomly allocated a random number from a random number table that was used as the seed for the computer-generated list of villages using SPSS software. The random selection of households for the entomological sampling was done in a similar way.

Due to the nature of the interventions, blinding of the study participants will not be possible, while observer bias will be reduced wherever possible. Microscopists shall read blood films blinded to the identity and intervention status of the subjects. By using standard light traps and exit traps, we shall reduce the mosquito collector bias. Moreover, the entomologists in our research group will examine the trap catches, which are different from the trap collectors.

### Interventions

#### Long-lasting insecticidal nets (LLINs)

The LLINs distributed for this trial were PermaNet 2.0 rectangular, 100 denier, light blue, family size (160 cm width x 180 cm length x 150 cm height) purchased in June 2014 from the Vestergaard Frandsen Group SA (Vestergaard Frandsen, Lausanne, Switzerland). The PermaNet 2.0 net is a factory-treated mosquito net manufactured with deltamethrin, which is expected to retain its biological efficacy for a minimum of 20 standard WHO washes or approximately 3 years under field conditions [53].

The *MalTrials* project bought a total of 10,000 PermaNet 2.0 LLINs for this trial. The nets were imported and arrived in the study site on 16 September 2014, and distributed to study households from 1 to 5 October 2014. All households in the LLINs + IRS and LLINs alone arms received new LLINs free of charge at the beginning of the intervention regardless of the previous ownership, with householders maintaining their existing nets at the time of distribution. The number of new LLINs distributed to each household was based on the household size recommended by the national malaria guidelines [43], i.e., one net for a family of 1–2, two nets for a family of 3–5, three nets for a family of 6–7 and four nets for a family of 8 or more people.

In advance of the LLINs distribution, all village residents were made aware of the distribution of the nets through house-to-house visits, village leaders and community elders. The net distribution was done in the center of the village based on a pre-determined registration list of households in each village by the *MalTrials* project personnel in collaboration with the district and village health workers. Households who did not receive the nets during the distribution schedule were identified and received their nets later. Education about, and a demonstration of how to use, LLINs were given to the recipients by trained field staff and selected village residents.

With an average of 2.57 nets per household, a total of 3006 households (1599 households in LLINs + IRS and 1407 households in LLINs only) in both arms of the trial received 7740 LLINs (4157 nets in LLINs + IRS and 3583 nets in LLINs only). The remaining nets were stored at an ambient temperature under safe storage conditions to be used for net replacement before the peak malaria transmission season each year (in August). Net use and retention at the household level are being monitored during the weekly household visit.

#### Indoor residual spraying (IRS)

Indoor residual spraying with propoxur will be carried out at three times during the study period in the LLINs + IRS and IRS alone arms. Spraying will be done once a year prior to the peak transmission season at the beginning, the middle and before the end of the trial, following the national spraying operation guidelines [43] and WHO operation manual guidelines [54]. Propoxur (isopropoxy-phenyl methylcarbamate) is highly effective against mosquito vectors for more than 3 months at a dosage of 2 g/m^2^ in the form of a water-dispersible powder (WP) [54], and will be acquired from the state-owned Adami Tullu Pesticide Processing Share Company located in the study district. Propoxur 50 % WP contains 2 g of active ingredient and is packaged in 400 g sachets, and two sachets will be mixed with 8 L of water. Preliminary findings using the WHO susceptibility tube tests in 2013 showed a 100 % susceptibility of *An. arabiensis* and *An. pharoensis* to propoxur (*MalTrials* unpublished pilot data).

We will use the average surface area measurement per unit structure (110 m^2^) to be sprayed by a spray man per day to calculate the correct amount of insecticide required for the spraying. All IRS operations will be carried out in collaboration with the DHO as per the recommendations of the national guidelines [43]. A 6-day training on spraying operation will also be given for locally recruited spray men and supervisors. The spraying teams will be organized by squads of four spray personnel and a porter, and supervised by a squad leader. The DHO malaria focal persons and HEWs will be used to organize, follow-up, and supervise the daily activity of spray teams, and a spray equipment and personnel protective clothing will be obtained from the DHO.

Approximately 12 houses will be sprayed by each spray operator per day using an 8-liter Hudson X-pert (HD Hudson Manufacturing Company, Chicago, IL, USA). Prior to spraying, a community sensitization will be performed to inform residents in regard to the safety, purpose and time of spraying. On the day of the IRS operation, all targeted households will be informed about the schedule, purpose and requirements of the spraying. The householders will be requested to prepare and vacate the house before spraying, and household items such as water, food and cooking utensils will be removed from the house. Household members will be allowed to enter the sprayed house after 30 minutes, and be requested to clean the floor and bury or burn the dirt. The householders will also be requested not to wash, paint or re-plaster the sprayed walls until at least the end of the main transmission season.

### Study endpoints

The primary health outcome measure is malaria incidence determined by the detection of *P. falciparum* or *P. vivax* by rapid diagnostic tests (RDTs) in patients with a fever or having a history of fever within the previous 48 hours upon arrival at health posts by passive case detection (PCD). The sensitivity and specificity of the RDTs compared with the results of the light microscopy will be determined. The secondary health outcome measure is mean Hb concentration in children under the age of 5 years, which is measured using a portable photometer (HaemoCue®, Ångelholm, Sweden) at the end of each transmission season through community-based house-to-house visits.

The primary outcome measure of the economic evaluation is the total cost of the interventions. The secondary outcome measures are the direct and indirect costs of the intervention from both the provider and societal perspectives, which includes Disability- adjusted Life Years (DALYs) and the number of malaria cases averted by each intervention.

The primary entomological outcome measure is malaria transmission expressed as EIR, estimated as the mean number of sporozoite infective bites/person/year. The secondary entomological outcome measures are the mean number of *An. arabiensis*/light trap/night, and mean number of *An. arabiensis* in spray catches/night inside houses.

### Sample size

#### Malaria incidence and anemia prevalence

Sample sizes were calculated based on unpublished epidemiological data collected in a baseline pilot study in villages adjacent to Lake Zeway during September to December 2013. The sample size for the primary endpoint, the incidence of malaria, was calculated using methods for cluster randomized trials [55] that take into account the intra-cluster correlation coefficient (ICC), incidence rate, the expected effect and the power of the study. Using a baseline malaria incidence rate of 7.85 per 10,000 person-weeks and the coefficient of variation between clusters within each group, we used *k* = 0.27 in the sample size estimation (*MalTrials* unpublished pilot data). Thirty-five households (approximately 175 people) per cluster will be followed up for 116 weeks, with 44 clusters achieving a 90 % power to detect a 25 % reduction in the malaria incidence rate in the LLINs + IRS arm compared to LLINs alone or the IRS only arm, using a two-sided 5 % significance level. We plan to follow approximately 1540 households with an estimated 7800 people in each arm of the trial (Fig. 2). Overall, the trial will cover over 31,000 people from approximately 6100 households. The proposed sample size is also assumed to suffice in terms of having the power to detect a mean reduction between the study arms of 0.5 mg/ml Hb concentration in children under 5 years of age.

#### Economic evaluation

The sample size calculated for malaria incidence is also assumed to suffice for the economic evaluation of the cost of interventions. The sample size for the cost of illness study (at the patient level) is calculated using a single population mean formula. Hence, 260 participants will be enrolled into the study from the passively detected malaria patients using a consecutive sampling technique.

#### Entomological study

We aim to calculate the sample size for the entomological collections based on the pilot entomological studies. In a West African study with a 90 % power and 5 % significance level, it was advised to sample eight houses from each of 15 clusters of the study arms, but the mosquito density was lower [56]. A total of 16 villages (four per arm) will be randomly selected for entomological study, in which indoor host-seeking mosquitoes will be collected by CDC light traps from four houses per arm, indoor resting mosquitoes from 16 houses per arm using pyrethrum spray collection and outdoor resting mosquitoes from four artificial pit shelters per arm of the study.

### Data collection methods

Each household will receive a specific identification (ID) number tagged onto a colored metal plate placed on the upper front door of the house. Each inhabitant in the household will also receive a unique personal, three-digit ID number (village number/household number/person number). The latitude and longitude of each household will be recorded using a hand-held Global Positioning System (GPS) device. The GPS data will be downloaded to a computer for the creation of maps of the study villages and for a spatial analysis of epidemiological, entomological and economic data. Guidelines for the data collections will be developed during the pilot studies. All questionnaires and forms will be initially prepared in English and translated into a local language for data collection.

#### Epidemiological data collections

PCD of malaria cases at the health posts will be carried out throughout the trial using RDTs, and thin and thick blood smears for microscopic examination. We will recruit 24 data collectors (two data collectors per *kebele*) for a weekly household visit, 14 nurses (one nurse per *kebele*) for malaria diagnosis and treatment at the health posts and three field supervisors. Data collectors will be trained on questionnaire specifics, interviewing techniques and household visits. They will visit households weekly to help identify residents with fever, or a history of fever in the last 48 hours, to refer to the health posts and track information on LLINs’ use by household members during the night before the survey.

The research team and supervisors will make regular field visits for quality control of the work done at health posts. Each field supervisor will be responsible for 59 clusters, and will meet with data collectors and health workers at the health posts at least once a week. During these visits supervisors will check patient registration books, malaria surveillance forms and the availability of antimalarial commodities, and collect completed forms and blood slides and bring them back to the project’s office. Throughout the study period, all health posts that participate in the trial will be provided with multispecies and mixed infection-detecting RDTs, artemether-lumefantrine (AL) and chloroquine obtained from the Oromia Regional Health Bureau (ORHB). The RDT that will be provided is the CareStart® Malaria *Pf/Pv* combo test (Access Bio, Inc., Somerset, NJ, USA), which is individually packaged with an alcohol swab, lancet, capillary tube, buffer and test device.

Through weekly household visits, study participants with a fever or having a history of fever within the past 48 hours, will be encouraged to present to the health posts. Residents will be advised to visit the health posts whenever they develop a fever. They will be asked to show a household card whenever visiting a health post. For individuals without a household card, health workers will use other information that allows them to locate the household and its number. Individuals who are found to be positive for *P. falciparum* by RDT will be given AL twice a day for 3 days based on body weight according to national guidelines [31]. AL is a fixed dose combination of 20 mg artemether plus 120 mg of lumefantrine. *P. vivax* positive individuals will be treated with chloroquine, 25 mg/kg for 3 days (10 mg base per kg on days 1 and 2, and 5 mg base per kg on day 3). Treatment of other conditions will be done in accordance with the national guidelines or a referral to higher level health facilities. Patients with severe illness at the time of visit (from malaria or other causes) will be referred to the nearest health facility. Any person from the study villages treated for malaria at a health center or hospital will also be included in the study.

Thin films of blood slides will be fixed with methanol for 30 seconds. Both thin and thick films will be stained with 3 % Giemsa for 20–30 minutes by experienced laboratory technicians in accordance with the standard malaria laboratory procedures. A thick blood smear will be declared negative with a minimum of 100 high power fields by microscopic examination, while the thin film will be examined to identify the *Plasmodium* species for positive slides. A second reading for all positive and negative slides will be performed by an experienced laboratory technologist and any discordant results will be resolved by a third reader. All blood slide readers will be blinded to the different arms of the trial and to the diagnosis of preceding readers.

At the end of the main malaria transmission season each year, the Hb concentration will be assessed in children 6–59 months old for assessing the prevalence of anemia using a portable photometer (HaemoCue®, Ångelholm, Sweden) in the field. Through house-to-house visits, a single finger-prick sample will be taken from each child, and children’s height and weight will also be measured. A Hb cut-off point of less than 11.0 g/dl will be used to decide whether a child has anemia, and will be further classified into mild, moderate and severe anemia.

Community-based malaria prevalence surveys will be conducted on a representative sample of households from each arm of the trial during the main transmission season (September to November) each year among all age groups. All household members will be eligible to be included in the study. Allowing for ineligible households, absence on the day of the survey and refusals at the household and individual level, approximately 5500 individuals from 1100 households will be estimated to be included in the study. This would provide an average 275 households from each arm of the trial to be included in the study. The households will be randomly selected from the total list of households in all arms of the trial. The heads of the households or their representatives will be interviewed using a pre-tested structured questionnaire. Voluntary individuals will be tested for malaria parasites using RDTs. Moreover, blood slides will also be collected for microscopic examination.

#### Economic evaluation data collections

Costing will be done based on the cost of the interventions from a health systems perspective, with cost data collected by interviewing individual malaria patients, family members and the DHO personnel. The main cost outcome measure will be the total costs of interventions, including all resources used to deliver the interventions and recurrent costs such as personnel, supplies and materials, the operation and maintenance of buildings, utilities and communication costs, in addition to capital costs such as buildings, equipment, and vehicles. The costing of the intervention will be done from the provider’s perspective using a standard malaria costing tool developed by the WHO [57]. The economic evaluation of health outcomes will be evaluated by the number of malaria cases averted and DALYs gained as a result of the interventions using incidence data. The number of deaths averted due to the interventions will be calculated based on the case fatality rates of malaria.

Economic evaluation, cost-effectiveness/utility analysis, presenting cost per malaria case prevented and costs per DALY averted will all be employed. The DALYs will incorporate both morbidity estimates from malaria episodes and anemia among children. We will develop a Markov life cycle model to account for the recurrent nature of malaria disease, with a varying amount of risk for repeated episodes during different seasons of the year. The cost of illness study will be conducted in the same district from non-trial villages to account for saved disease treatment costs. Epidemiological and effectiveness parameters will be based on the findings of this research project, while the clinical course of the diseases will be based on a review of the best available literature. Standard DALY weights will be utilized. Moreover, this trial will incorporate an evaluation of the distributional impact of the interventions using inequality analysis techniques and a decomposition of the inequality into various socioeconomic factors using an extended cost-effectiveness analysis (ECEA). In the ECEA, we will estimate the benefit of the interventions across different levels of income group in terms of the number of deaths prevented, the net financial risk protection provided, the out-of-pocket expenditure averted and the number of poverty cases prevented.

#### Entomological data collections

The results of trials from countries where *An. gambiae* s.s. is the dominant vector might not be fully applicable in countries such as Ethiopia, where *An. arabiensis* is the main vector. The latter vector is less affected by mosquito nets, and is more exophilic and less anthropophilic [58]. Estimating the human biting rate (HBR) of mosquitoes is important for a risk assessment of malaria transmission. The Centers for Disease Control and Prevention (CDC) light traps, used in many studies, are only a proxy measure to estimate HBR. Human landing catches (HLC) are a more direct way to estimate HBR, and are often considered the “gold standard” [59]. Recent research showed that light trap collections placed close to inhabited mosquito nets may not be a reliable method of assessing human biting rates [60].

The CDC light traps and HLC will be compared before starting the trial. Since HLC cannot be used for community-wide mosquito collections due to ethical and logistical drawbacks, CDC light trap collections will be calibrated with HLC locally in order to estimate an operational conversion factor for calculating the HBR and EIRs. Mosquito collections will be carried out during the malaria transmission season following interventions.

The CDC light traps, pyrethrum spray sheet catches and artificial pit shelters will be employed to collect mosquitoes and assess their biting behavior, indoor and outdoor resting densities. Houses will be randomly selected from a computer-generated list and the person will be blinded to the arms and houses. For CDC light traps and pyrethrum spray catches, the number of houses will be four and 16 from each arm, respectively, while four pit shelters in each arm will be constructed. Mosquitoes will be collected weekly from August to November each year, and will be identified to species using a morphological key [61]. Previous studies indicated the presence of *An. arabiensis* as the only species of the *An. gambiae* complex in the study area [50]; however, for reconfirmation purpose a species-specific polymerase chain reaction (PCR) will be applied [62].

From all collections, blood-engorged female mosquitoes will be analyzed using Enzyme- linked Immunosorbent Assays (ELISAs) for determining the blood meal source of vectors in indoor or outdoor situations [63]. All collections of female anophelines, with the exception of blood-fed mosquitoes, will be subjected to a sporozoite test by employing ELISA [64] and a parity determination by ovary dissection [65].

Insecticide resistance in *An. arabiensis* and *An. pharoensis* will be monitored annually throughout the study period using standard WHO tube tests [66]. The insecticides will be the pyrethroids (deltamethrin, alphacypermethrin, permethrin and lambdacyhalothrin) and the carbamates (bendiocarb and propoxur). In order to assess any change in resistance, the resistance intensity will be quantified. For the insecticide susceptibility tests, larvae and pupae of the two species will be collected from breeding habitats and reared to adults. The status of physiological susceptibility/resistance of females will then be determined. Molecular [67] and biochemical analyses [68] will be used to identify potential insecticide resistance mechanisms. Sporozoite rates will be determined by ELISA and rechecked by real-time PCR [69]. The decay rate of propoxur will also be assessed monthly by conducting cone wall bioassays on eight randomly selected houses from the two arms (IRS and LLINs + IRS) [70] for a period of at least 6 months post spraying. An insectary colony of *An. arabiensis* (Debre Zeit strain, being maintained at the Aklilu Lemma Institute of Pathobiology, Addis Ababa University since 2001), which is susceptible to all insecticides including propoxur, will be used for the bioassay test.

### Data management

Data will be collected using standardized paper-based forms and questionnaires according to standardized operating procedures. Data will be entered into a computer by trained data entry clerks, we will verify data by range and consistency checks, and data cleaning will be done weekly. Any discrepancies will be corrected by cross-checking against the corresponding original forms and subsequently amended in the final dataset.

All blood slides will be labelled with the patient’s unique ID number and date of collection to help ensure anonymity. To minimize any loss to follow-up, we will keep following up all residents and maintain their database, even if they move out of the trial area or move from one cluster to another cluster with a different intervention. For residents or respondents who are not present at the time of the visit by project staff, basic information about dates and reasons for absence will be sought from other community members such as friends or neighbors. The epidemiological data, economic evaluation data and entomological data will all be kept separately. All databases will be password protected and only accessed by research team and data entry clerks for data entry, cleaning and analysis. Furthermore, data will be stored for at least 5 years and be made publically available.

Each of the principal investigators of the epidemiological, economic evaluation and entomological studies will maintain records in compliance with Good Clinical Practice (GCP) for regulatory or institutional requirements. Authorized representatives from the funding agency, ethical committees or regulatory bodies may inspect all documents and records of the trial. The research team will explain any deviation from the originally approved protocol. Moreover, any deviation from the protocol that will have an impact on the conduct of the study will also be immediately reported to the funding agency and the local Institutional Review Board (IRB) as appropriate.

### Analytical plan

The primary health outcome measure is malaria incidence determined by the detection of *P. falciparum* or *P. vivax* using RDTs. All analyses will be conducted on an intention-to-treat basis, regardless of whether the individual household members use LLINs, IRS or not. Malaria cases diagnosed with an infection within 28 days of the first episode with the same *Plasmodium* species will be censored and will not be included in the analysis. An analysis will be performed as a community randomized trial with time-person as the denominator. All analyses will be conducted using Stata version 13 (StataCorp LP, College Station, TX, USA), and primary and secondary outcomes will be compared between the different intervention arms and control groups. The main outcome variable, malaria incidence based on PCD, is assumed to follow a Poisson distribution based on a random and independent occurrence. Hence, a generalized Poisson log linear model will be fitted to measure for associations between the outcome variable and predictors. The main outcome variable will also be analyzed as a binary variable and will be compared in the intervention and control clusters using multilevel mixed-effects logistic regression models, taking into account the clustering effects. In addition, we will also analyze the data using the population as the denominator (e.g., with a generalized estimating equation (GEE), multilevel analysis or spatial analysis) to help assess the effect of the intervention.

To control for potential confounding factors, the clustering effect of villages, the effect of repeated measurement in the same individual and individual level covariates (such as age, gender, LLINs’ use) will be taken into consideration during the analysis. Other potential confounding factors will also be adjusted for in the regression analysis, and all estimates will be presented with 95 % confidence intervals. Time will be included as a fixed effect that will allow any interaction with the interventions to be quantified.

The WHO age-adjusted cut-off for Hb will be used to classify anemia in children [71]. For children between 6–59 months of age, a normal Hb level is defined as Hb of 11.0 g/dl or greater and as mild at 10.0–10.9 g/dl. Moderate anemia is defined as children with an Hb level of 8.0–10.9 g/dl while severe anemia is defined as an Hb level of less than 8.0 g/dl. At a community level, a prevalence of anemia will be stated to be severe if over 40 % of the children are anemic (combining mild, moderate and severe) and moderate if the prevalence is 20–39.9 %. We will measure the weight and height of all children under the age of 5 years, and calculate the anthropometric indices such as weight for height, height for age, and weight for age. Both malaria and anemia prevalence data will be compared in the intervention and control clusters using multilevel mixed-effects logistic regression models, taking clustering effects into account.

Cost-effectiveness, expressed as an incremental cost-effectiveness ratio (ICER), will be calculated for each outcome and arm using standard DALY weights. The interventions will be ranked according to cost-effectiveness, whereas inequality in terms of health outcomes, will be measured by the Gini coefficient and the concentration index [72].

The agreement between the two mosquito collection methods, HLC and light traps, in assessing mosquito sampling efficiency will be calculated by a parametric approach based on an analysis of variance and simple graphical methods. Indoor and outdoor *Anopheles* densities will be compared among the study arms using a one-way analysis of variance (ANOVA) if data are normally distributed or using a Mann-Whitney *U* test if data are non-normally distributed.

### Ethical considerations

#### Ethical approval

The study was approved by the IRB of the College of Health Sciences at Addis Ababa University, the Ministry of Science and Technology in Ethiopia (ref: 3.10/446/06) and the Regional Committee for Medical and Health Research Ethics, Western Norway (ref: 2013/986/REK Vest). The protocol was registered online on 8 September 2014 at the Pan African Clinical Trials Registry under the registration number PACTR201411000882128.

#### Community consultation and sensitization

Prior to the implementation of interventions, a consultative workshop and several meetings were held to explain the objectives, *kebele* selection and randomization, implementation procedures and expected outcomes of the trial to the communities with representatives from the ORHB, the East Shewa Zone Health Department and the Adami Tulu District Administration. Permission through official letters was obtained from various administrative levels. Study communities were sensitized prior to randomization through meetings and discussions with community leaders, *kebeles* and village leaders and community elders.

#### Information and informed consent

Verbal informed consent to participate in the study was obtained beforehand from the study participants and from parents/guardians for children under 18 years of age using the local *Afan* Oromo language. Information sheets were provided to inform about the purpose of the study, and the participants were informed that involvement in the study was voluntary and that they had the right to withdraw at any time regardless of reason. At each data collection, the verbal consent of the study participants and verbal assent from the parents/guardians for children were obtained using the local language. Assurance was also given that a refusal to participate in this study would not affect their access to services at the health posts in the study villages in the community.

#### Adverse events and malaria treatment

We do not anticipate any physical harm or risks to the participants. Blood samples for RDTs, microscopic examination of slides and Hb measurements will be collected using aseptically disposable lancets. A finger-prick for blood sample collection may result in mild pain and bruising at the site where blood is obtained, but will not cause any further harm. The collection of blood samples from finger-pricks is part of the routine procedures in the diagnosis of malaria by health workers. Malaria treatment will be provided according to the national guidelines using AL and chloroquine, which are the first-line antimalarial treatments for *P. falciparum* and *P. vivax*, respectively [43]. All study participants visiting health posts will be examined and treated for malaria free of charge, while the mass distribution of LLINs and IRS spraying will be carried out by the *MalTrials* project for free in collaboration with the DHO. Mosquito collectors will be trained to collect mosquitoes as soon the mosquitoes land and before they bite. To help minimize risk, data collectors will be provided with an appropriate prophylactic drug (Malarone) before the collections.

#### Confidentiality of information

To the best of our ability, all information from the study households and participants will be held in confidence and appropriate measures will be taken to ensure the confidentiality of information both during and after data collection. Access to information will be limited to data collectors, including health workers and their supervisors at sites of collection, data entry clerks and to the research team.

#### Trial oversight

There is no need for a Data Safety and Monitoring Board (DSMB) for this trial since all interventions, blood sample collection and treatments are part of routine malaria control in Ethiopia and will be undertaken in collaboration with the health workers at the health posts and the DHO. We do not foresee any adverse effects from the interventions, so we do not intend to apply any stopping rules for this trial. No participant in the control group will be impeded from obtaining mosquito nets from other sources, and malaria incidence in the control villages will be monitored throughout the study for possible case build-up or an outbreak of malaria. If the control villages encounter any malaria outbreaks, an intervention will be taken by the *MalTrials* project and the DHO in accordance with the national guidelines regardless of the trial.

Deltamethrin-treated LLINs and propoxur IRS are WHO recommended and meet the specifications of the WHO’s Pesticide Evaluation Scheme, and our interventions will follow the WHO and national recommendations [44, 73]. The hazard which may be associated with this trial is that the insecticides used for the IRS could leak into the environment. Empty sachets, cartons, plastic bags, used gloves, pricking needles and other contaminated materials will be handled properly until they are finally burned. Lastly, all needle safety procedures will be in line with WHO standard.

### Timelines of activities

The trial is being carried out for a period of approximately 116 weeks from September 2014 to December 2016, with Table 1 showing details of the timetable of activities.

**Table 1:** Timetable of activities

Activity by year and month	J	F	M	A	M	J	J	A	S	O	N	D
2013												
Ethical approval		x	x	x	x	x	x	x	x	x		
Development of data collection tools					x	x	x	x	x	x	x	x
Census and mapping			x	x								
Epidemiological pilot study									x	x	x	x
Entomological pilot study						x	x	x	x	x	x	
2014												
Selection of clusters		x	x	x	x							
Randomization of study clusters						x						
Procurement of LLINs			x	x	x	x	x	x				
Procurement of insecticide				x	x	x						
Household numbering and tagging						x	x					
Baseline data collection							x	x				
LLINs distribution									x	x		
Protocol registration								x	x			
IRS spraying									x			
LLINs distribution									x			
PCD and weekly household visit									x	x	x	x
Entomological surveys							x	x	x	x	x	
Cost data collection									x	x	x	x
2015												
Anemia survey	x											
Malaria prevalence survey									x	x	x	
PCD and weekly household visit	x	x	x	x	x	x	x	x	x	x	x	x
Entomology lab assays and field surveys	x	x	x	x	x	x	x	x	x	x	x	x
Cost data collection	x	x	x	x	x	x	x	x	x	x	x	x
IRS spraying and LLINs’ replacement								x				
Data entry and cleaning	x	x	x	x	x	x	x	x	x	x	x	x
Sensitization of study population								x	x	x		
2016												
Anemia survey	x											x
Malaria prevalence survey									x	x	x	
PCD and weekly household visit	x	x	x	x	x	x	x	x	x	x	x	x
Entomology lab assays and field surveys	x	x	x	x	x	x	x	x	x	x	x	x
Cost data collection	x	x	x	x	x	x	x	x	x	x	x	x
IRS spraying and LLINs’ replacement								x				
Data entry, cleaning and analysis	x	x	x	x	x	x	x	x	x	x	x	x
Entomological lab assays and field surveys	x	x	x	x	x	x	x	x	x	x	x	x
Cost-effectiveness analysis	x	x	x	x	x	x	x	x	x	x	x	x
2017												
Dissemination of findings to the study community	x	x										
Data analysis and report writing	x	x	x	x	x	x						
Scientific writing and publications	x	x	x	x	x	x	x	x	x	x	x	x
Final report to the funding agency				x	x	x						
Dissemination of findings to stakeholders							x					

*LLINs* long-lasting insecticidal nets, *PCD* passive case detection

## Discussion

In an effort to accelerate the reduction and ultimate elimination of malaria, IRS with insecticide and the universal distribution of LLINs have been implemented in recent years in many countries in SSA. It is well-accepted that decisions regarding malaria interventions should be based on robust evidence of the benefits and cost-effectiveness of the interventions. Since the rollout of both interventions requires considerable resources, there is an urgent need to evaluate additional protective benefits and the cost-effectiveness of the combination of the interventions. This study aims to measure whether IRS in combination with LLINs increases protection against malaria incidence compared to the use of LLINs alone, IRS alone or current routine practices. The intervention will consist of four “arms”: LLINs + IRS, LLINs alone, IRS alone and control (routine practice).

The main outcome of the trial will be the assessment of the incidence of malaria using PCD at health posts. Our sample size is relatively large compared to other trials, e.g., a study in the Gambia used 35 clusters of 110 children in each of two arms to detect a 50 % reduction in the incidence of malaria [56]. Our preliminary sample size calculations based on malaria incidence rates in southern Ethiopia [60] showed that we might need to include 10 clusters per arm with approximately 50 households (250 persons) per cluster. However, a Cochrane review advocates large-sized clusters [8].

Conducting research to evaluate the impact of community interventions raises a number of practical issues [21]. Both LLINs and IRS interventions are implemented by the project, and we anticipate that the coverage of the interventions will be very high despite issues of net use and the re-plastering of sprayed walls of the houses. We understand that the recent relatively low incidence of malaria in the area will have an impact on the proper and consistent use of LLINs by the community, which may challenge an active participation by the community. We will monitor LLINs ownership and use by household members on a weekly basis, and we will also assess the magnitude of the re-plastering rates of houses and teach the community about its consequences on malaria.

Some of the strengths of our study are that we have carried out extensive pilot studies before starting the trial. This enabled us to estimate the variance in malaria incidence among the villages and thus the sample size. In the previous published trials, the IRS insecticide was bendiocarb which has a relatively short residual duration on the walls, or DDT, against which the insecticide resistance has become widespread [38–42]. The current trial evaluates the added protection of propoxur, which has a relatively longer duration on walls compared to DDT. In addition, we also determined that the anopheline vectors in the study area are susceptible to propoxur. One of the limitations of this trial is that the communities will not be masked or blinded to the intervention. This would create a bias towards an increased effect of the combined intervention compared with other arms of the trial. This trial will not use a buffer zone between clusters of the different interventions as it will be carried out in rural areas with scattered households. As a result, the proximity of the clusters may influence the results. However, this applies to only some of the selected villages that are close to each other. As the positions of all households are recorded, we shall also try to adjust for possible close proximity of the villages during analysis.

The community in the “routine practice” with a weekly follow-up is in a better position than the community in the “routine practice” without any follow-up. Therefore, we decided to allow the trial with all four arms and emphasized that every participant will have access to weekly visits by the *MalTrials* project field personnel, early diagnosis and state of the art treatment for malaria. Adami Tullu was one of the 19 IRS targeted districts of the President’s Malaria Initiative (PMI) and the Ministry of Health (MOH) between 2008 and 2013 [74]. However, since 2014 the PMI has shifted its target areas to other districts in the Oromia Region. However, we will continuously monitor all groups including the control arm of the study if unforeseen malaria epidemics should arise.

This study is proposed at a time of significant need within the countries of SSA on how to effectively use IRS and LLINs interventions for malaria control and elimination. Consequently, the study is well-timed to assess whether the combination of LLINs and IRS could contribute towards the elimination of malaria. The trial addresses how to promote the uptake of research findings into public health programs by enhancing the knowledge base on interventions that will improve the effectiveness and coverage of anti-malaria interventions.

This study is expected to generate important evidence to inform the malaria control programs and the public regarding the effectiveness and cost-effectiveness of the two major vector control interventions. The costs of our intervention is similar to that of the existing malaria control program so that they can be easily accepted by the MOH. We anticipate that the findings of this study will be used for an effective planning and implementation of vector control interventions.

### Trial status

At the time of the submission of this manuscript, ethical approval has been obtained and the trial had completed a pilot study, baseline data collection and randomization, and is ongoing.

## Abbreviations

AL: artemether-lumefantrine
ANOVA: analysis of variance
CDC: Center for Disease Prevention and Control
CONSORT: Consolidated Standards of Reporting Trials
DALYs: Disability-adjusted Life Years
DDT: dichloro-diphenyl-trichloroethane
DHO: District Health Office
DSMB: Data Safety and Monitoring Board
ECEA: extended cost-effectiveness analysis
EIR: entomological inoculation rate
ELISA: Enzyme-linked Immunosorbent Assay
GCP: Good Clinical Practice
GEE: generalized estimating equation
GPS: Global Positioning System
Hb: hemoglobin
HBI: Human Blood Index
HBR: human biting rate
HEW: health extension worker
HLC: human landing catches
ICC: intra-cluster correlation coefficient
ICER: incremental cost-effectiveness ratio
ID: identification
IRB: Institutional Review Board
IRS: indoor residual spraying
ITNs: insecticide-treated nets
LLINs: long-lasting insecticidal nets
MOH: Ministry of Health
ORHB: Oromia Regional Health Bureau
PCD: passive case detection
PCR: polymerase chain reaction
PMI: President’s Malaria Initiative
RDT: rapid diagnostic test
SPSS: Statistical Package for Social Sciences
SSA: sub-Saharan Africa
WHO: World Health Organization
WP: water-dispersible powder
